# Land use in urban areas impacts the composition of soil bacterial communities involved in nitrogen cycling. A case study from Lefkosia (Nicosia) Cyprus

**DOI:** 10.1038/s41598-021-87623-y

**Published:** 2021-04-14

**Authors:** Coralea Stephanou, Michalis Omirou, Laurent Philippot, Andreas M. Zissimos, Irene C. Christoforou, Slave Trajanoski, Anastasis Oulas, Ioannis M. Ioannides

**Affiliations:** 1 Department of Agrobiotechnology, Agricultural Research Institute, Nicosia, Cyprus; 2 Université Bourgogne Franche-Comté, INRA, AgroSup Dijon, Agroécologie, 21000 Dijon, France; 3 Geological Survey Department, Ministry of Agriculture, Rural Development and Environment, Nicosia, Cyprus; 4 Center for Medical Research, Medical University of Graz, Graz, Austria; 5 Cyprus Institute of Neurology and Genetics, Bioinformatics Group, Engomi, Cyprus; 6 Department of Agrobiotechnology, Agricultural Microbiology Laboratory, Agricultural Research Institute, Athalassa, Cyprus

**Keywords:** Ecology, Microbiology

## Abstract

The different types of land-use and soil lithology in urban and peri-urban areas of modern cities compose a complex mosaic of soil ecosystems. It is largely unknown how these differences result in changes in bacterial community composition and structure as well as in functional guilds involved in N cycling. To investigate the bacterial composition and the proportion of denitrifiers in agricultural, forested, schoolyard and industrial areas, 24 samples were collected from urban and peri-urban sites of Lefkosia. Bacterial diversity and the proportion of denitrifiers were assessed by NGS and qPCR, respectively. Proteobacteria, Actinobacteria, Bacteriodetes, Chloroflexi, Acidobacteria and Planctomycetes were identified as the most dominant phyla across all sites, while agricultural sites exhibited the highest bacterial diversity. Heavy metals such as Co, Pb, V and Al were identified as key factors shaping bacterial composition in industrial and schoolyard sites, while the bacterial assemblages in agricultural and forested sites were associated with Ca. Variance partitioning analysis showed that 10.2% of the bacterial community variation was explained by land use management, 5.1% by chemical elements due to soil lithology, and 1.4% by sampling location. The proportion of denitrifiers varied with land use management. In industrial and schoolyard sites, the abundance of the *nos*ZII bacterial community increased while *nir*K abundance declined. Our data showed that land use and lithology have a moderate impact on the bacterial assemblages in urban and peri-urban areas of Lefkosia. As the *nos*ZII bacterial community is important to the N_2_O sink capacity of soils, it would be interesting to elucidate the factors contributing to the proliferation of the *nos*ZII clade in these soils.

## Introduction

The world’s growing population requires increasingly more land for physical infrastructure and resources, forcing the expansion of urban settlements on the peripheries of cities. Global urban land cover is expected to increase by more than 200% by 2030 with major challenges for urban sustainability^1^. The consequences of urbanisation come at multiple scales and include loss of fertile agricultural land^2^, habitat fragmentation and biodiversity reduction^3,4^, modified hydrological systems, soil contamination and local climates^5^, and changes in energy and biogeochemical cycles^6,7^.

Urban and peri-urban ecosystems are characterized by spatio-temporal changes in land use, with previous studies reporting potentially adverse effects of urbanisation on soil ecosystems^8^. The increased variability in the physico-chemical properties of urban and peri-urban soils, including agricultural and forest lands, is mainly attributed to soil compaction^9^, irrigation and nutrient inputs^10–12^, heavy metals accumulation^13^, the deposition of oxides and other organic compounds from increased road traffic^14,15^, and the presence of different plant species^16^.

Schools, private and public green spaces, private gardens, industrial and agricultural activities as well as forested areas are composing a complex mosaic pattern of soils in urban and peri-urban areas with different land-use management and history. It is largerly unknown how these land-use differences in an urban and peri-urban environment result in changes in bacterial community composition and structure. Recent studies reported that urbanization could affect the composition and function of soil microbial communities^17–20^, implicating urban construction and development as a major driver of change in bacterial composition and the α-diversity of bacterial communities^21,22^. Anthropogenic activities, chemical contaminants, and the amount of water applied in green infrastructures have also been shown to affect the diversity and function of soil bacterial communities^23,24^. On the other hand, Ramirez et al.^25^ showed that the diversity of bacterial guilds was similar between soils collected from the New York Central Park and a “global soil” sample set, while the study by Xu et al.^8^ suggested that the bacterial diversity found in urban parks of China was not influenced by urbanization-related factors. Such conflicting results highlight the complexity of factors influencing the bacterial community structure in urban and peri-urban areas.

Despite a significant research output on the diversity and composition of bacterial communities in urban areas, the study of microbial communities participating in nitrogen (N) cycling in urban soils remains limited^19^. Nitrogen is an essential nutrient for the productivity and the fertility of terrestrial ecosystems with the many different N-transformations being controlled by the various functional microbial communities. Within the N-cycle, denitrification comprises the respiratory reduction of soluble nitrate and nitrite into gaseous forms, thus becoming the main process responsible for the return of fixed nitrogen to the atmosphere. This process has received considerable attention towards efforts to mitigate climate change since it is a major source of nitrous oxide (N_2_O), which is both a potent greenhouse gas and the dominant ozone depleting substance after the suppression of CFCs by the Kyoto Protocol. The key steps of this process are catalyzed by the nitrite reductase encoded by *nirK* or *nirS* and the N_2_O reductase encoded by *nosZ*I or *nosZ*II. Changes in land use might impact the composition of microbial communities related with soil-N cycling in rural areas. For example, it has been suggested that disturbed sites encompass more denitrifiers compared to sites outside the urban environment^17^. Moreover, the abundance of *nosZ*II genes was higher in suburban turfgrass sites compared to the abundance measured in urban turfgrass sites^19^. Recently, Deeb et al.^24^ suggested that green infrastructure sites in urban environments provide significant levels of microbial biomass and activity that are important for carbon (C) and N cycling.

This study aimed to identify key drivers of bacterial community composition in soils from different land use types in the urban and peri-urban area of Lefkosia, Cyprus. It builds on a previous detailed survey of the city’s soil geochemistry with focus on four typical and distinct types of urban land use, namely schoolyards, forested areas, industrial and agricultural production areas. Specific aims sought to examine (1) how the abundance and composition of the total bacterial community are influenced with either geology or land use and (2) whether differences in the soil geochemical characteristics are affecting the abundance of community N-cycling microorganisms with a focus on N_2_O-producing and -reducing bacterial communities.

## Material and methods

### Study area, sampling sites, and physicochemical properties

**Figure 1:**
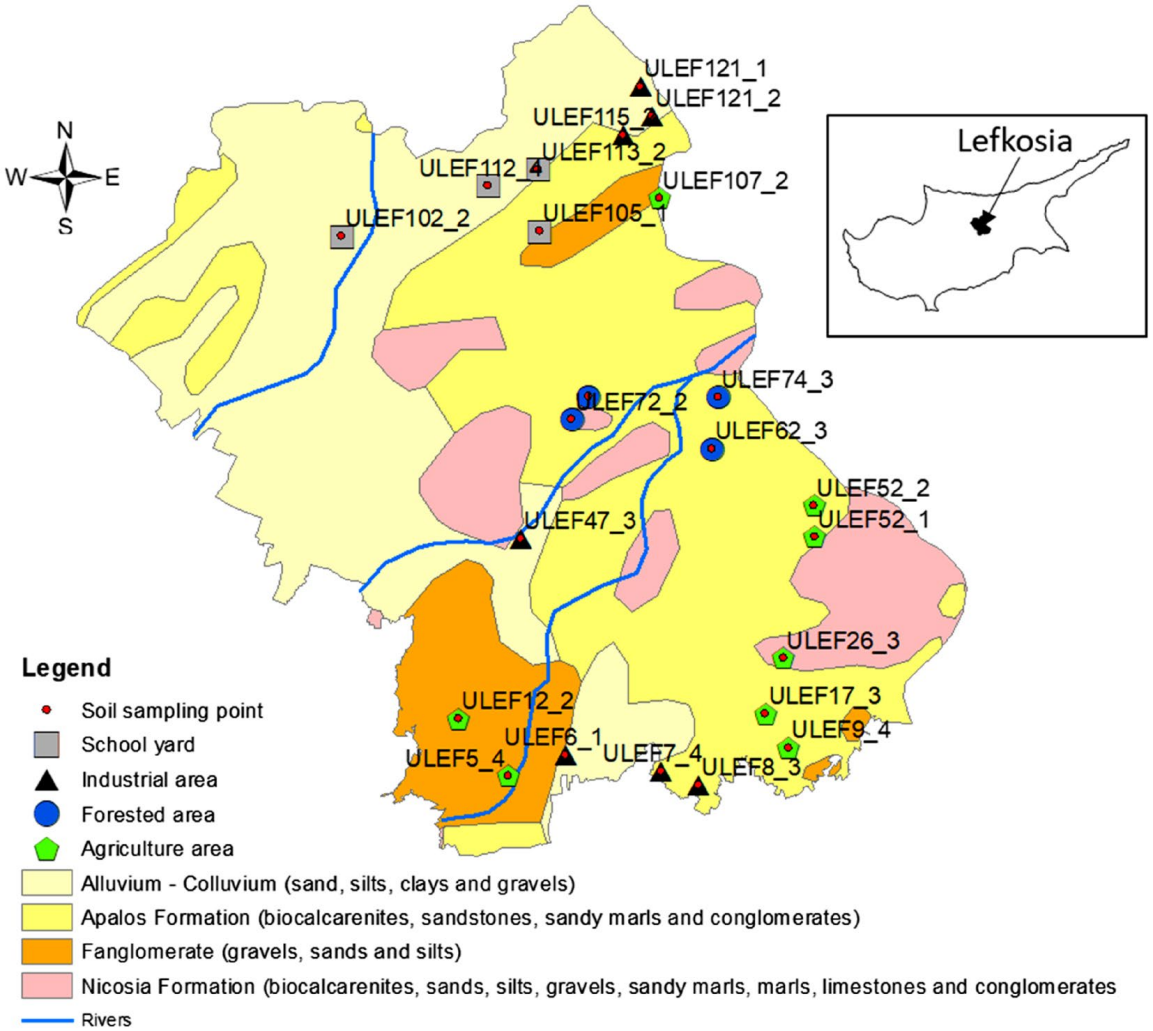
Sample sites analysed for bacterial composition in soils of Lefkosia with land use for each sample. Geology shown as background. Map was prepared using ArcGIS Desktop 10.8 (http://www.esri.com).

Soil samples were obtained from the metropolitan area of Lefkosia, the capital of the Republic of Cyprus. The city of Lefkosia remains a divided city in Europe since the 1974 occupation by Turkish military forces. Land use within the city is patchy with unused space intertwined with urban and commercial areas. Industrial activity is characterized as light with small scale manufacturing and processing mainly within designated industrial sites. On the outskirts of the city there are large spaces of cultivated land (agricultural) mainly with seasonal crops and olive trees, as well as forested areas that are used as parks and recreation areas. The geology of the region consists of carbonate-rich lithologies, as shown in Fig. 1. Sampling was carried out using a regular grid dividing the area into 1 × 1 km^2^ cells. Samples were taken near the surface, approximately at a 10–20 cm soil depth, as four 250 g subsamples from the edges of a 5 × 5 m^2^ rectangle. Composite samples of 1-kg mass were sieved to 2 mm in the field and split in the laboratory for different treatments. A sieved subsample was stored at − 80 °C for DNA extraction and molecular analysis. Additional information about the study area, soil sampling procedures and the geochemical characteristics of the Lefkosia sample set have recently been published by Zissimos et al.^26^, where a total of 441 soil samples were analysed for 53 major and trace elements following a hot aqua regia digestion protocol. In the present study, 24 sampling sites from the total set of samples were selected to represent all types of land use. The selection was based on the lithology and the spatial distribution of the sites in the urban and peri-urban areas of Lefkosia (Fig. 1). These included plots of school property (e.g., schoolyards), industrial areas, both free of vegetation, agricultural ecosystems of *Olea europea*, and forested areas dominated with *Pinus silvestris* within the urban center. The forested areas cover over 800 hectares of green spaces and woods amidst the surrounding urban settlements, representing an example of natural land use with no visual impact of human activity (Fig. 1 and Supplementary Fig. 1).

### Physicochemical soil parameters and carbon measurements

#### Determination of pH

A portion of 5 g of sample was transferred to a beaker and mixed with 25 ml of deionised water. The suspension was stirred for 15 min. The pH and EC was measured using a Mettler Toledo Seven Compact pH meter and WTW inoLab Cond7310 EC meter, respectively.

#### Determination of total carbon (TC) and total organic carbon (TOC)

Total carbon (TC) was measured on 5 g of an air dried soil using an Eltra CS-800 automatic Carbon–Sulfur (CS) analyser. In parallel, 5 g of soil was transferred in a crucible and placed in a high temperature furnace at 500 °C for 4 h to remove organic carbon, and then analysed to the CS analyser for the determination of inorganic carbon (IC). Total organic carbon calculated by subtracting IC from TC.

### Soil DNA extraction

DNA was extracted from approximately 0.3 g soil per sample using the DNeasyPowerSoilKit (QIAGEN, Germany) according to the manufacturer’s instructions. DNA concentration was measured on Quantus fluorometer (Promega, USA) and DNA quality was assessed by reading absorbance ratios at 230, 260 and 280 nm on Nanodrop ND-2000 (Thermo Scientific, USA). The extracted DNA was stored at − 20 °C for further processing. Only DNA with 260:280 and 260:230 ratios of 1.8 and 1.5, respectively, was used in further analyses.

### Quantification of total bacteria and bacterial denitrifier abundance

The abundance of total bacteria and of denitrifiers was quantified by quantitative PCR (qPCR) with SYBR Green chemistry using 16S rRNA, *nirK*, *nirS*, *nosZ*I and *nosZ*II genes as molecular markers according to Henry et al.^27^, Bru et al.^28^ and Jones et al.^29^. Quantitative PCR was performed on the CFX96 real-time PCR detection system with the CFX Manager Software v3.1 (Bio-Rad, USA). Reactions were performed in a total of 20 μL volume containing 10 μL of iTaq Universal SYBR Green Supermix (Bio-Rad, USA), 200 nM (16S rRNA), 1 μM (*nirK*, *nirS*, and *nosZ*I) or 2 μM (*nosZ*II) of forward and reverse primers (Microsynth AG, Switzerland), and 5 μL of 1 ng DNA template. No-template controls were included into every assay, giving null or negligible values. The purity of qPCR products was checked by melt curve analysis. Standard curves were obtained using tenfold dilution series (10^8^ to 10^2^ copies/μL) of linearized plasmids containing the targeted gene. The standard curve was constructed using four points diluted standards with R^2^ values higher than 0.99, slopes ranging from − 3.748 to − 3.923 and reaction efficiencies from 81.1 to 86.7%. The presence of PCR inhibitors in the DNA extracted from soil was estimated by (i) diluting the soil DNA extract and (ii) mixing a known amount of standard DNA with soil DNA extract prior to qPCR. No inhibition was detected in either case. These conditions are reported in accordance with the minimum information for publication of quantitative qPCR experiments^30^. Data were expressed as the number of gene copies per gram dry soil. For comparisons between different soil types, the abundance of denitrification genes was normalised to the total bacterial abundance by calculating the ratio of gene copies between denitrification and 16S rRNA genes.

### Illumina-sequencing of the 16S rRNA gene

The 16S rRNA gene V3-V4 variable region PCR primers 515/806 with barcode on the forward primer were used. Sequencing was performed on a MiSeq following the manufacturer’s guidelines. Sequence data were processed using the QIIME2 2019.7 analysis framework (Caporaso et al., 2010) integrated in Galaxy^31^ of the Medical University Graz cluster (BioMedNode). The raw reads were quality-filtered, de-noised, de-replicated, merged and checked for chimeras using DADA2 pipeline^32^ with standard settings followed by taxonomic assignment with QIIME2 sklearn-based classifier against SILVA rRNA database Release 132^33^ at 99% identity. Phylogenetic tree was created with FastTree^34^ on Mafft^35^ aligned representative sequences. Data was uploaded to ENA (European Nucleotide Archive), supported by EMBL-EBI, with primary Accession PRJEB41189.

### Statistical analysis

Statistical analyses and figures were performed using RStudio (Version 1.2.1335). The absolute OTU reads were divided by known 16S rRNA gene copy numbers from bacterial genomes obtained from the rrnDB database version 5.7 including 20,225 Bacteria records, representing 5716 species (available at https://rrndb.umms.med.umich.edu/static/download/)^36–38^. For bacterial genera without reported gene copy number, unidentified bacteria and other bacteria that are not represented in the database, the OTUs reads were devided by 1.8 (average 16S rRNA copy numbers from the database)^37^. The unidentified bacteria and those genera that were not present in the database were also devided by 1, without detectable differences in downstream analysis. Hellinger transformation was used to normalise feature abundance table using the deconstand function of the vegan package. Non-metric dimensional scaling (NMDS) analysis based on the Bray–Curtis dissimilarity matrix was performed to evaluate differences between soils with different land-use characteristics. The homogeneity of group dispersion was evaluated with the betaspider function of the vegan package. PERMANOVA and ANOSIM were applied using the *adonis* and *anosim* functions of the vegan package to test for statistically significant differences in bacterial compositions of soil samples with different land-use. Correlation between soil chemical properties and bacterial composition was performed using the envfit function, and Redundancy Analysis (RDA) was implemented to reveal whether soil chemical parameters could explain the variation of bacterial guilds in soils with different land-use. Indicator analysis based on genera was performed using the multpatt function with 999 permutations, allowing combinations of samples from different land-use using the indicspecies package^39^. The analysis is based on the exclusivity and the fidelity of the species in the different habitats and an indicator values that is calculated^40^. The indicator value determined, reflects the association between a species and a habitat and its significance is tested using permutation test^39^.One-way ANOVA was conducted on 16S rRNA-normalised gene copy numbers of *nirK*, *nir*S, *nosZ*I and *nosZ*II genes at the 95% confidence level using Tukey HSD to determine whether different land uses affect their presence in soil. In the case of a non-normal distribution of residuals, the non-parametric Kruskal–Wallis test was implemented to assess the effect of land-use on the examined soil element, followed by post-hoc Dunn test to reveal discrepancies between soils with different land-use. Spearman correlation was performed between soil chemical properties and the relative abundance of 16S rRNA and the four denitrifying genes . The 16S rRNA gene copy number was log10-transformed. All analyses were performed after outlier removal.

## Results

### Bacterial and denitrification functional gene abundance

**Figure 2:**
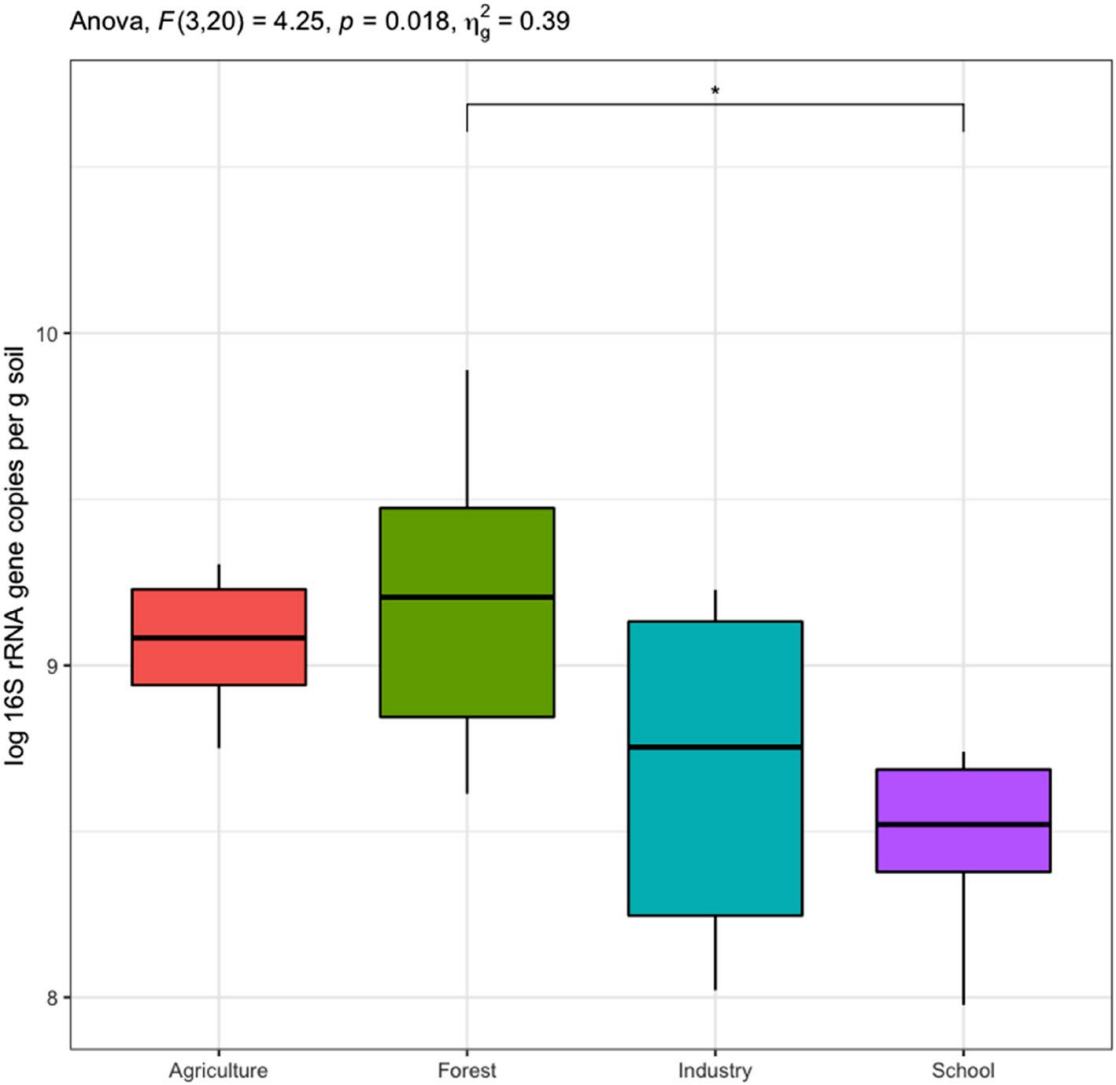
Log transformed 16S rRNA gene copy numbers per g of soil in samples derived from agricultural, industrial, forested and schoolyard areas. Spreads in the boxplots denote standard error of the mean and asterisks show statistically significant differences between treatments (*p < *0.05*).

**Figure 3:**
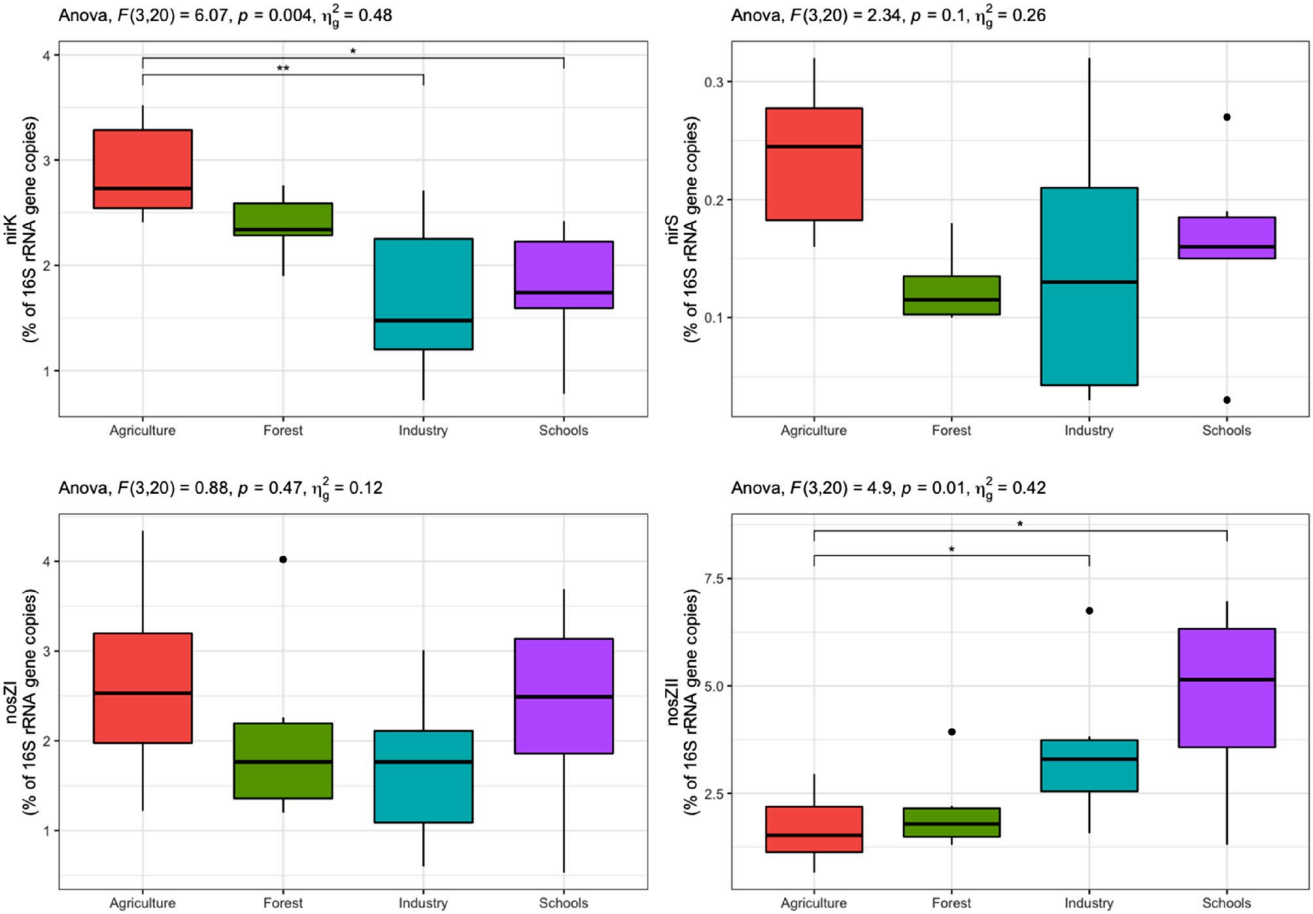
The %age abundance of *nir*K, *nirS*, *nosZ*I and *nosZ*II genes normalised to 16S rRNA gene abundance in soil samples derived from agricultural, industrial, forested and schoolyard areas. Spreads in the boxplots denote standard error of the mean and asterisks show statistically significant differences between treatments (**p < *0.01* and *p < *0.05*).

A qPCR method was used to assess the abundance of the total soil bacterial community based on 16S rRNA gene copy numbers. In addition, *nirK*, *nirS*, *nosZ*I, and *nosZ*II denitrification gene copy numbers were used as proxies for the abundance of the denitrifier community to assess differences in relative abundances of all detected denitrifierying community in the different land-use types. Findings showed that soil land-use management did not affect the abundance of the total bacteria community (F_(3,18)_ = 1.28, p = 0.31). Specifically, 16S rRNA gene copy numbers ranged from 9.5 × 10^7^ to 7.7 × 10^9^ per gram of dry soil with samples from forested and agricultural areas having marginally higher abundance (Fig. 2). The relative abundance of the bacterial denitrifying community varied significantly across soils from different land use types (Fig. 3). Notably, the abundance of the *nirK* bacterial community was substantially higher in agricultural soils compared to forest and school playground soils (Fig. 3), whereas the *nosZ*II bacterial community was higher in schoolyards and industrial soils compared to agricultural soils. The abundance of bacterial communities harbouring the *nir*S or *nosZ*I genes was similar across the different land-use management practices (Fig. 3).

### Bacterial community composition and diversity

In the current study, we implemented high-throughput sequencing to explore the bacterial community composition of soils with different land-use management. After filtering low-quality sequences and chimeras, we obtained 4,939,292 high-quality sequence reads. These reads were assigned to 42 Phyla, 125 Classes, 201 Orders, and 574 Genera. The most abundant taxa included (Supplementary Fig. 2) *Proteobacteria* (ranged from 24.9 to 30.7%), *Actinobacteria* (ranged from 18.2 to 22.3%), *Bacteriodetes* (ranged from 9.6 to 12.8%), *Choloroflexi* (ranged from 6.8 to 10.1%), *Planctomycetes* (ranged from 5.9 to 9.7%), *Acidobacteria* (ranged from 4.3 to 10.4%) and *Gemmatimonadetes* (ranged from 3.9 to 5.8%). Land-use management was shown to have a significant effect on *Planctomycetes*, *Gemmatimonades* and *Acidobacteria* (Supplementary Fig. 3). The relative abundance of *Planctomycetes* was similar between agricultural and forested soils, and was substantially lower in sampling sites from schoolyards and industrial plots. The relative abundance of *Gemmatimonadetes* was higher in agricultural soils than in industrial plots and schoolyards, and *Acidobacteria* were higher in agricultural and forested soils than in soils from industrial plots. Also, there was a trend of higher abundance of *Proteobacteria* and *Bacteroidetes* in industrial plots compared to soils derived from agricultural areas, but pairwise comparisons did not show significant differences (Supplementary Fig. 3).

**Figure 4:**
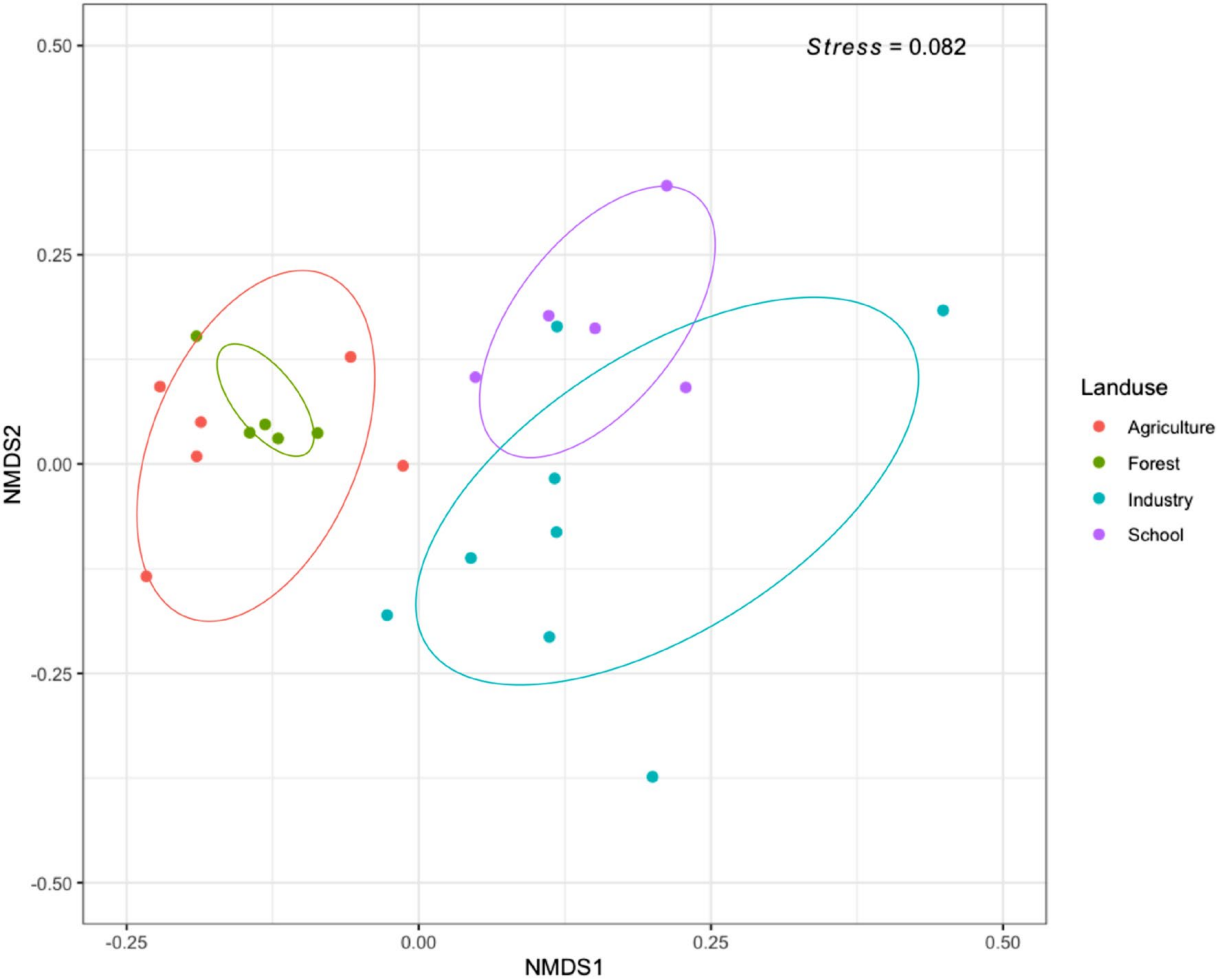
Non metric dimensional scaling plot showing Bray–Curtis distance of 16S rRNA abundance bacterial communities. Distance between the community composition obtained from agricultural (red circles), forested (green circles), industrial and schoolyard (purples circles) sites. The ellipses represent 95% confident intervals.

Non-metric dimensional scaling on weighted unifrac distances showed that the microbial communities from different samples could be grouped according to the four land-use categories (Fig. 4). Ordination results were further supported by non-parametric analysis of similarities (ANOSIM) and permutational multivariate analysis of variance (PERMANOVA) based on Brays-Curtis dissimilarity. The ANOSIM analysis showed significant separation of bacterial assemblages by land-use (R = 0.53, *p* = 0.012), with similar results obtained after PERMANOVA analysis (F model = 22.73, R^2^ = 0.39, *p* = 0.0017). The homogeneity of the bacterial communities was examined by measuring the distance between the centroids of each site (F = 2.67, *p* = 0.29), suggesting a homogenous dispersion of the several features found in the different land-use, and a higher variation in industrial sites.

### Representative microbial features of the different land-use soil samples

**Figure 5:**
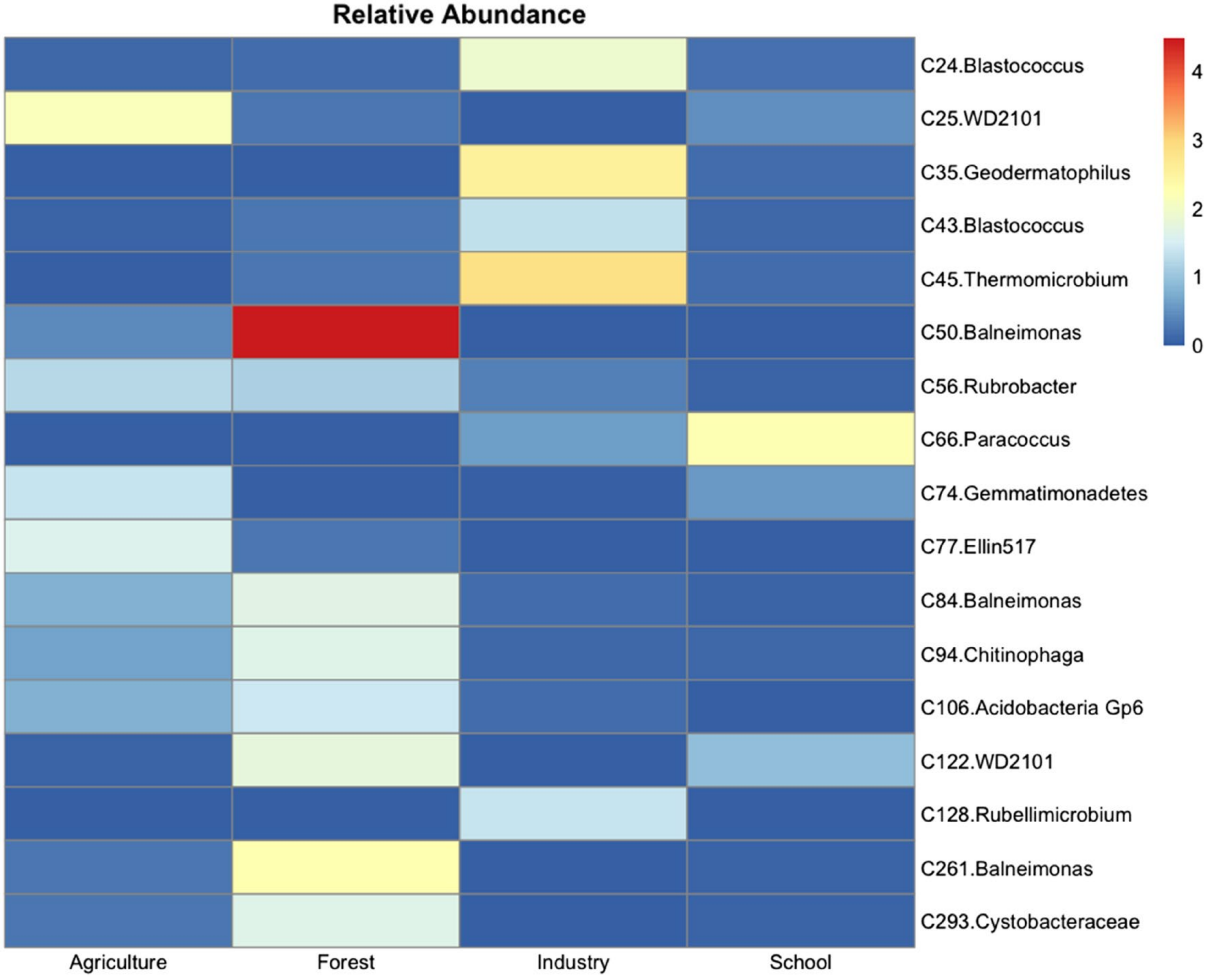
Heatmap of the relative abundance of the different OTUs exhibiting significantly (*p adj* < *0.05*) high indicator values (Inv > 0.5) and high abundances (> 1% relative abundance) compared with mean relative abundance in each land-use soil. Indicator analysis was performed using the indicspecies R package.

Species indicator analysis was implemented to identify bacterial taxa significantly associated with particular ecological habitats. Out of a total of 155 taxa (with relative abundance greater than 1%), 17 had a significantly high indicator value (> 0.5). Specifically, 14 taxa were associated with one site, three with two sites (Fig. 5), but none was identified to be associated with all the sampling sites. Forest and agricultural land-use sites shared two common indicator taxa that were assigned to the genus of *Rubrobacter, Acidobacteria Gp6*. In schoolyards, two features were identified as indicators, one of which was assigned to *Paracoccus*, and an unassigned *Gemmatimonadetes*. In industrial sites, two taxa were assigned to *Blastococcus,* one to *Thermomicrobium* and one in an unassigned genus of *Geodermatophilaceae*. In agricultural soils, three features were identified as indicators, including *Phycisphaera‐*like planctomycetes WD2101, one taxa assigned to Ellin517 and an unassigned *Gemmatimonadetes*. In forested sites, three taxa were assigned to *Belneinomas*, one to an unassigned *Cystobacteraceae* and one to the *Phycisphaera‐*like planctomycetes WD2101 soil group*.*

### Relationships between environmental variables, denitrifiers and microbial community composition

**Figure 6:**
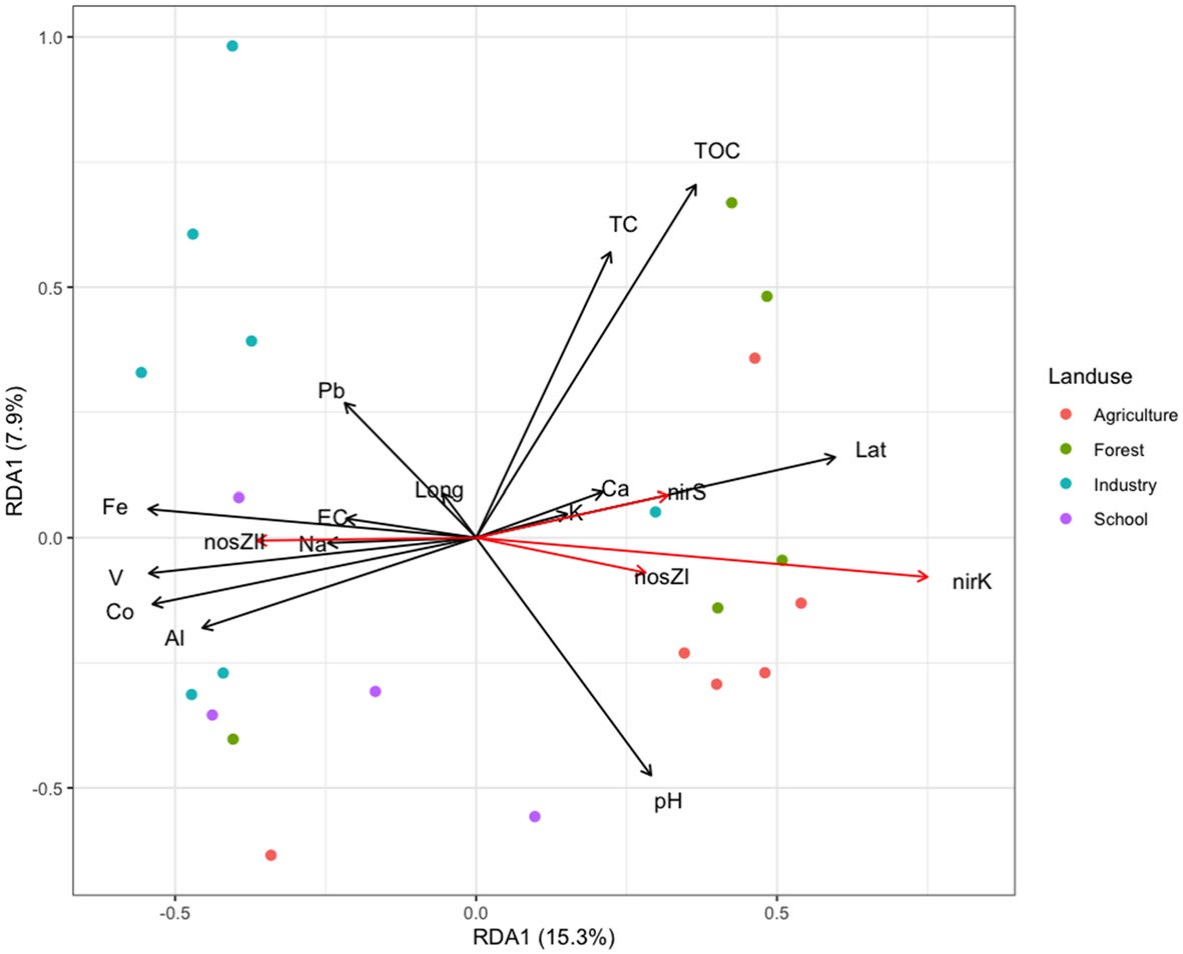
Redundancy analysis (RDA) of soil bacterial communities at genus level. Plots were generated using Euclidean distance matrices with 9999 permutations using Vegan package. Black colored vectors represent environmental variables, which were forward selected (*P*_*adj*_ ≤ *0.05*). Red colored vectors represent *nir*K, *nir*S, *nos*ZI and *nos*ZII normalized to 16S rRNA abundance from soils with different land-use management.

Redundancy analysis was implemented to characterize relationships between bacterial community composition, environmental parameters and denitrifier community abundance. Based on the forward selection algorithm of Vegan package, ten chemical variables, namely pH, TOC, EC, Ca, Fe, Na, K, Co, Pb and V were identified as significant variables that could explain variation in bacterial community composition (Fig. 6). In addition to the chemical elements, the spatial distribution of sampling sites also had a significant effect on the community composition (F = 4.22, *p* = 0.032). Specifically, along the first RDA axis (F = 5.73, *p* = 0.016), two groups were separated, mainly determined by the chemical elements. Industrial and schoolyard sampling sites clustered closely at the negative values of RDA1 axis and were mainly determined by Al, Co, Pb, V, Fe and Na, whereas agricultural and forested sampling sites were clustered at the positive values of RDA1 and were mainly determined by pH, TC, TOC, Ca and K. Geographical factors (longitude and latitude) contributed significantly to the variance (Fig. 6), while lithology classes did not affect bacterial community composition. The bacterial denitrifying community was significantly associated with the composition of the bacterial community in the different land-use sampling sites. The abundance of *nosZ*II bacterial communities was associated with industrial sampling sites in the negative values of RDA1 axis, while the remaining of the bacterial communities were related to agricultural and forest samples in the positive values of RDA 1 axis.

**Figure 7:**
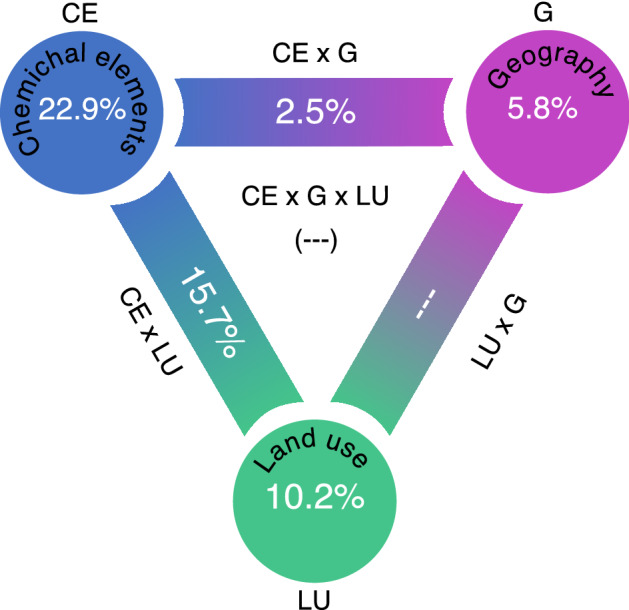
Variation partitioning analysis of microbial community explained by chemical elements characteristics (CE), land-use (O), and geographic location (G). Diagram represents the biological variation partitioned into the relative effects of each factor or a combination of factors. The edges of the triangle represent the variation explained by each factor alone. The sides and the middle of the triangles represent interactions of the factors.

The total contribution of chemical characteristics, land-use characteristics and geographic location to explaining bacterial community variance amounted to 38.9% as depicted in Fig. 7. Chemical elements, owing to the chemical properties of the parent soil, explained 22.9% of the total bacterial variance, while land-use management and georgraphic location explained 10.2% and 5.8%, respectively (Fig. 7). The interactions between land-use and chemical characteristics and between chemical characteristics and geographic location explained 15.7% and 2.5% of the variance in community composition, respectively. The interaction between land-use and geographic location, and a three-way interaction with all variables both had a low impact on bacterial community composition.

## Discussion

Here, we investigated the bacterial composition, diversity and abundance at urban and peri-urban areas of Nicosia, the capital of the Republic of Cyprus. Microbial communities are of tremendous importance due to their capacity to perform soil functions that underpin soil-based ecosystem services. The most abundant taxa identified in the current study were *Proteobacteria* (ranged from 24.9 to 30.7%) , *Actinobacteria* (ranged from 18.2 to 22.3%), *Bacteriodetes* (ranged from 9.6 to 12.8%), *Choloroflexi* (ranged from 6.8 to 10.1%), *Planctomycetes* (ranged from 5.9 to 9.7%), *Acidobacteria* (ranged from 4.3 to 10.4%) and *Gemmatimonadetes* (ranged from 3.9 to 5.8%). These findings are in accordance with previous studies performed in urban and peri-urban environments, suggesting that the dominant phyla in the bacterial communities are constant^8,19,25^. The industrial and schoolyard soils had a significantly lower abundance of *Planctomycetes*, *Acidobacteria* and *Gemmatimonatedes* compared to agricultural and forested sites. However, the differences noticed were Phylum dependent. (Supplementary Fig. 1). Published work showed that heavy metal contamination in soils impacts bacterial community, reporting a decrease in the abundance of *Planctomycetes* under high Pb and Zn concentrations^41^, and of *Acidobacteria* and *Gemmatimonas* in heavy metals contaminated soils^42^. Exposure to heavy metals could enhance the selection of tolerant taxa and induce the adaptation of the bacterial community via several mechanisms, including biosorption increased activity, chelation, bioprecipitation, and extracellular precipitation^43,44^. As to identify individual OTUs sensitive to specific land-use management sites, we implemented indicator species analysis. Notably, we did not identify any taxa to be associated with all sampling sites. In schoolyards, indicator taxa were assigned to *Paracoccus*, while in industrial sites, indicator taxa were assigned to *Thermomicrobium* and *Geodermatophilus*. Members of these taxa include bacteria that are able to grow in biotopes, which are commonly characterised by poor and compacted (anaerobic) soils, dry and heated soils, as well as soils polluted with heavy metals^45,46^. Both the schoolyards and industrial sites were overlaid with a similar transported basaltic fill (Supplementary Fig. 2), which is the main material used with naturaly elevated chemical elements^26^. The compression of this material at these sites and the absence of plant cover can reduce oxygen levels and increase the temperature of soils, thereby promoting the growth of microorganisms that are able to proliferate under these conditions.

Previous studies suggested that soil disturbance caused by land-use changes and management can lead to a substantial reduction in soil microbial diversity^47,48^. Industrial soils exhibited the lowest α-diversity, which could be associated with the elevated levels of some heavy metals and possibly other pollutants found in this type of soils. The highest bacterial diversity was detected in soils from agricultural areas (Table 1), likely because agricultural management practices can change the soil habitat through tillage, nutrient inputs (organic or inorganic) and pesticides. The increase of bacterial diversity in agricultural soils in this study could be the result of the combined effect of these practices on the bacterial community. It was previously stated that intermediated disturbance may lead to an increase in soil bacterial diversity when soil effective resources are not limiting^49^. Moreover, the incorporation of above-ground plant biomass during winter in these fields, together with nutrients addition and soil tillage, may lead to an increase in soil bacterial diversity due to a rearrangement of the spatial context of microbial communities and their resources^50^.

**Table 1:** Comparison of bacterial α-diversity indexes among different land-use management samples.

Land-use	Observed	PD	Chao1	ACE	Shannon	Fisher
Agriculture	1788.4 a	122 a	1796.1 a	1802.7 a	7.1 a	396.1 b
Forest	1351.6 b	107 a	1357.4 b	1363.0 b	6.3 b	314.6 ab
Industrial	1153.2 b	82 b	1158.5 b	1161.6 b	6.4 b	243.8 a
School	1457.0 b	91 b	1564.2 b	1367.9 ab	6.5 b	409.3 ab
Land-use effect	*p* = *0.038*	*p* = *0.021*	*p* = *0.012*	*p* = *0.015*	*p* = *0.047*	*p* = *0.028*

Different letters indicate statistically significant differences between the means at *p* < *0.05.*

The composition of bacterial communities in the urban and peri-urban areas of Lefkosia were grouped according to the land-use management and were correlated to the soil chemical properties and the geography of the sampling sites (Figs. 4 and 6). Redundancy analysis showed that the samples from forested and agricultural land-use plotted along the first axis, exhibiting dependencies on soil geochemistry of mainly carbonate lithologies, pH, TC and TOC (Fig. 6). Samples obtained from schoolyards were plotted alongside the industrial samples, presumably because both sites were overlaid with similar transported fill materials of mid-brown to grey colour. These materials are typical of basaltic-derived soils in Cyprus, which are used as fills on many of the industrial sites of the country. The presence of human transported material was shown to have a significant effect on the bacterial community composition of New York urban soils, which was attributed to changes in water movement and chemical properties^51,52^. The chemical properties explaining the variance of bacterial community composition in schoolyards and industrial sites were V, Co, Pb, Fe, Na and Al, some of which were previously shown to characterize these sites^26^. Indeed, Hemmat-Jou et al.^53^ showed that an increase in the soil concentration of Pb and Zn had a negative effect on both α- and β-diversity of bacterial species. Similarly, increased Co levels were highly toxic, affecting the bacterial community in soils^54^. In addition to the chemical composition of soils, samples showed a higher dependency on longitudinal differences rather than latitude-orientated differences, which could be an artifact attributed to one or both of the following factors; (1) the way the city of Lefkosia is constructed along an axis of expansion that is forced by the continued division of the city, and (2) the longitudinal rather than lateral diversity in underground lithology. Based on variation partitioning results, 38.9% of the total variance of the bacterial community could be explained by soil chemical properties, land-use management and the geography of the region where samples were collected. Additionally, pH and total organic C have been identified as important factors shaping bacterial composition in soils under different land-use management. Previous studies documented that soil pH as well as total organic carbon could be among the most important soil chemical properties shaping bacterial communities^55–57^. Also, other factors such as soil water-retention, soil texture, temperature and nutrients availability could also affect bacterial composition in soils under different land-use management^58,59^.

Previous work showed that the abundance of denitrifiers across landscapes was explained mostly by soil chemistry, to a lesser extent by spatial distance, and with a neglibible contribution by land management^57^. This response was attributed to the distribution of soil parent material and to the soil chemistry, and is in line with the findings of the current study. Shifts in bacterial community composition in schoolyards and industrial sites were related to changes in the proportion of denitrifiers. The proportion of bacteria harboring *nosZ*II was 2.7 times higher in industrial sites and schoolyards than in agricultural and forested sampling sites. The *nirK*-bacteria were 1.7 times more abundant in agricultural soils than in industrial sites and schoolyards. On the contrary, the proportion of *nir*S as well as *nosZ*I denitrifiers was similar across all sites (Fig. 3). The differential response between *nirK* and *nirS* denitrifiers in the current study further supports the general assumption that the two communities occupy different ecological niches^60,61^. Our RDA analysis showed that the direction of the *nosZ*II vector was different from that of the other denitrification genes (Fig. 7), suggesting that a different community is harboring this gene. Studies using intergenomic comparison Graf et al.^62^ showed that approximately 51% of the microorganisms with *nosZ*II do not carry the *nirS* and *nirK* genes, while more than 90% of the *nosZ*I bacteria have either *nirS* or *nirK*. Understanding the potential drivers of the *nosZ*II community is of particular environmental concern due to the role of this community in curbing N_2_O emissions from soil^63–65^. Here, the *nosZ*II community was enriched in the samples from industrial areas, which are characterized by well compacted soils owing to the presence of transported basaltic fills that were compressed by mechanical means. Whether a reduction in oxygen levels or other physico-chemical properties in these soils was a selective factor for the *nos*ZII bacterial community warrants further studies.

The present study showed that land use management altered the composition of bacterial communities likely via anthropogenic soil transport mechanisms in schoolyards and industrial areas. Soil managed under agricultural practices exhibited the highest bacterial diversity. Heavy metals such as Co, Pb, V and Al were identified as an important factor shaping the bacterial composition of industrial and schoolyard soils, while pH and Ca were the nutrient correlated the most to bacterial assemblages in agricultural and soil carbon (organic and inorganic) for forest samples. Moreover, the proportions of denitrifers also varied with land-use management but did not correlate with any of the soil chemical properties measured. Due to their role in the soil N_2_O sink capacity, it would be interesting to understand that factors influencing the *nos*ZII community in industrial and schoolyard soils.

## Supplementary Information

Supplementary Information
