# Supplementary material for: Land use in urban areas impacts the composition of soil bacterial communities involved in nitrogen cycling. A case study from Lefkosia (Nicosia) Cyprus
Source: Sci Rep. 2021 Apr 14;11:8198. doi: 10.1038/s41598-021-87623-y (PMC8047022; doi:10.1038/s41598-021-87623-y)

**Land use in urban areas impacts the composition of soil bacterial communities involved in nitrogen cycling.A case study from Lefkosia (Nicosia) Cyprus.**

Coralea Stephanou ^1^, Michalis Omirou ^1*^, Laurent Philippot^2^, Andreas M. Zissimos ^3^ Irene C. Christoforou^3^, Slave Trajanoski ^4^, Anastasis Oulas^5^, Ioannis M. Ioannides ^1^

^1^ Department of Agrobiotechnology, Agricultural Research Institute, Nicosia, Cyprus

^2^ Université Bourgogne Franche-Comté, INRA, AgroSup Dijon, Agroécologie, 21000 Dijon, France

^3^ Geological Survey Department, Ministry of Agriculture, Rural Development and Environment, Nicosia, Cyprus

^4^ Center for Medical Research, Medical University of Graz, Graz, Austria

^5^ Cyprus Institute of Neurology and Genetics, Bioinformatics Group, Cyprus

**Supplementary Figure 1.** Characteristic sites of land use selected for bacterial extraction and examination in the soils of Lefkosia. Clockwise from upper left forested area (ULEF74_3), industrial area (ULEF115_2), agricultural area (ULEF09_04), schoolyard (ULEF102_2). Codes are representing soil sample coding of the sampling sites.

**Supplementary Figure 2** Average relative abundances (%) of bacterial community assemblies at phylum level found in soil samples derived from agricultural, industrial, forested and school playground areas


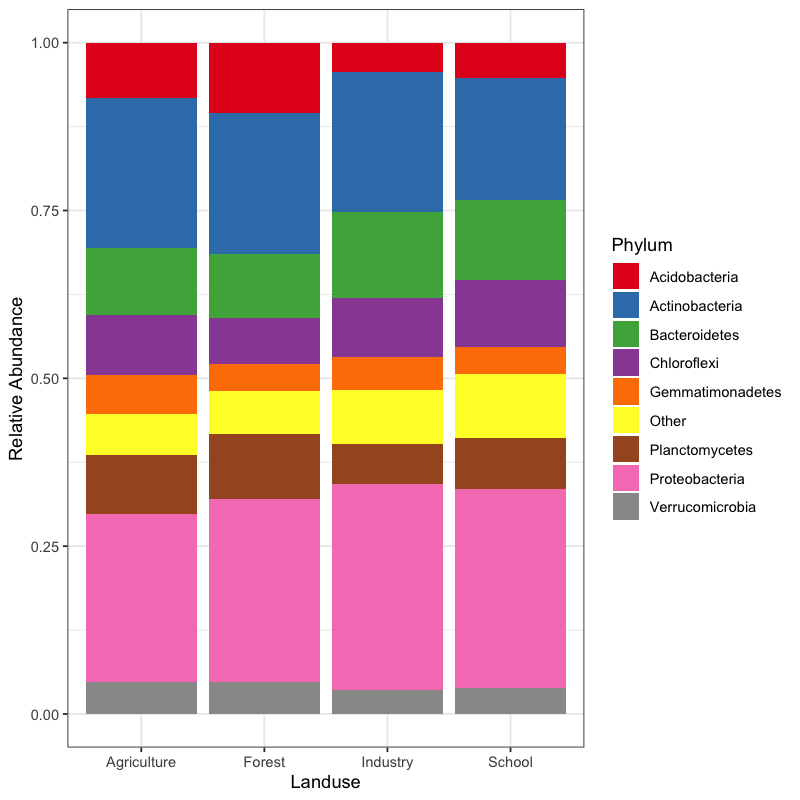


**Supplementary Figure 3** Relative abundances (%) of bacterial community assemblies at phylum level found in soil samples derived from agricultural, industrial, forested and school playground areas. Spreads in the boxplots denote standard error of the mean and asterisk are showing statistically significant differences between treatments (****, *p<0.001,* ***, *p<0.005,* **, *p<0.01* and *, *p<0.05*)


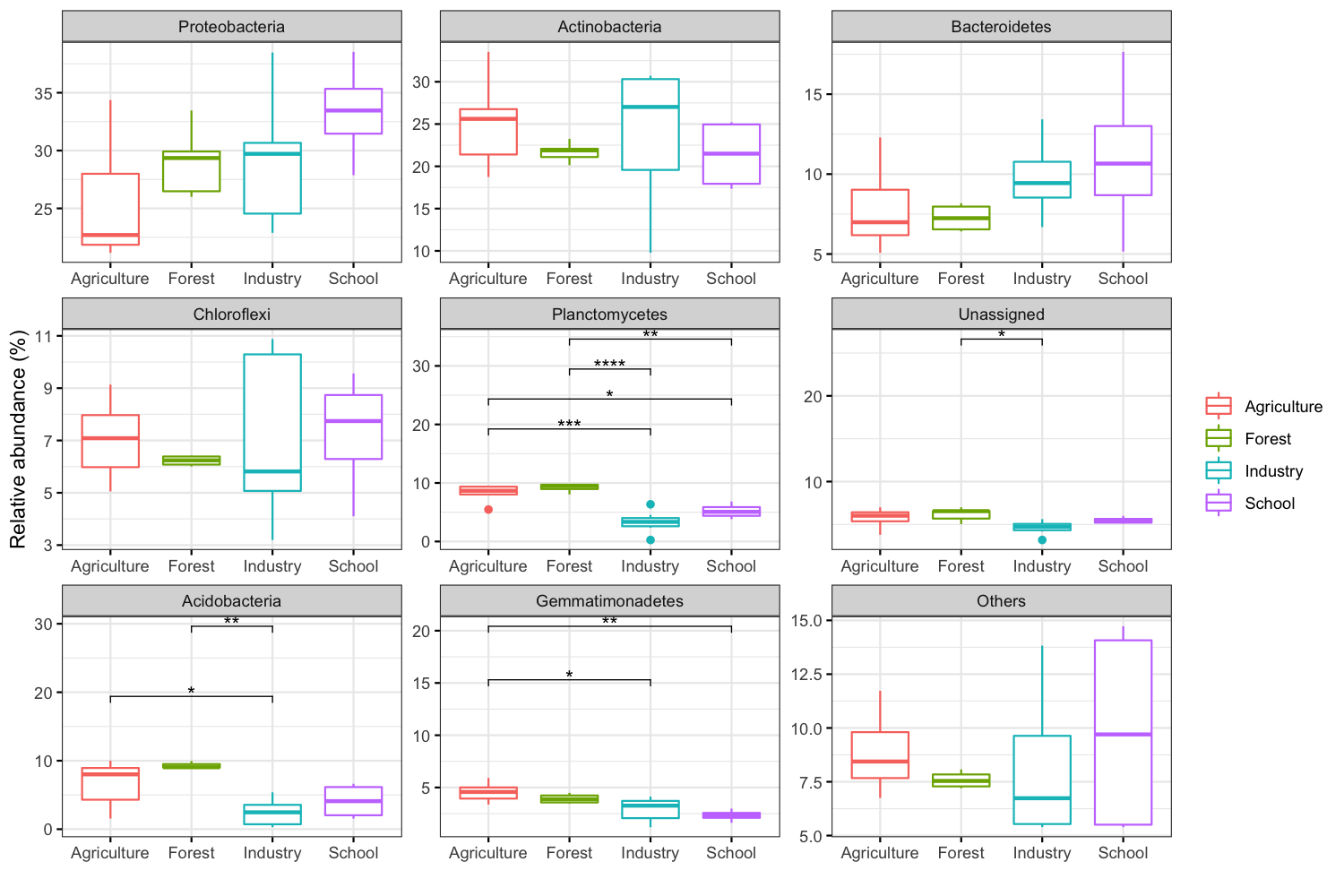

Supplement: Supplementary file 1 — Supplementary Information [file 41598_2021_87623_MOESM1_ESM.docx]
